# Nutrition Mediates the Relationship between Osteosarcopenia and Frailty: A Pathway Analysis

**DOI:** 10.3390/nu12102957

**Published:** 2020-09-27

**Authors:** Justin Chew, Audrey Yeo, Suzanne Yew, Cai Ning Tan, Jun Pei Lim, Noor Hafizah Ismail, Wee Shiong Lim

**Affiliations:** 1 Department of Geriatric Medicine, Tan Tock Seng Hospital, Singapore 308433, Singapore; Jun_Pei_LIM@ttsh.com.sg (J.P.L.); noor_hafizah@ttsh.com.sg (N.H.I.); wee_shiong_lim@ttsh.com.sg (W.S.L.); 2 Institute of Geriatrics and Active Ageing, Tan Tock Seng Hospital, Singapore 308433, Singapore; audrey_jp_yeo@ttsh.com.sg (A.Y.); Suzanne_PY_YEW@ttsh.com.sg (S.Y.); Cai_Ning_TAN@ttsh.com.sg (C.N.T.); 3 Department of Continuing and Community Care, Tan Tock Seng Hospital, Singapore 308433, Singapore

**Keywords:** nutrition, osteosarcopenia, frailty

## Abstract

Osteosarcopenia is associated with increased risk of adverse outcomes such as falls and fractures. Its association with frailty is less well-described, particularly in independent community-dwelling older adults. Although nutrition plays a crucial role in maintaining bone and muscle health, the complex relationship between osteosarcopenia and nutrition in the pathogenesis of frailty remains to be elucidated. In this cross-sectional analysis of 230 independent, community-dwelling individuals (mean age 67.2 ± 7.4 years), we examined the associations between osteosarcopenia with nutritional status and frailty, and the mediating role of nutrition in the association between osteosarcopenia and frailty. Osteosarcopenia was defined as fulfilling both the Asian Working Group for Sarcopenia 2019 consensus definition (low relative appendicular skeletal muscle mass adjusted for height, in the presence of either of either low handgrip strength or slow gait speed) and T-score ≤ −2.5 SD on bone mineral densitometry. We assessed frailty using the modified Fried criteria and nutrition using the Mini-Nutritional Assessment. We performed multiple linear regression, followed by pathway analysis to ascertain whether nutrition mediates the relationship between osteosarcopenia and frailty. Our study population comprised: 27 (11.7%) osteosarcopenic, 35 (15.2%) sarcopenic, 36 (15.7%) osteoporotic and 132 (57.4%) normal (neither osteosarcopenic, sarcopenic nor osteoporotic). Osteosarcopenia (β = 1.1, 95% CI 0.86–1.4) and sarcopenia (β = 1.1, 95% CI 0.90–1.4) were significantly associated with frailty, but not osteoporosis. Nutrition mediated the association between osteosarcopenia and frailty (indirect effect estimate 0.09, bootstrap 95% CI 0.01–0.22). In conclusion, osteosarcopenia is associated with frailty and poorer nutritional status, with nutrition mediating the association between osteosarcopenia and frailty. Our findings support early nutritional assessment and intervention in osteosarcopenia to mitigate the risk of frailty.

## 1. Introduction

Osteoporosis and sarcopenia are two important musculoskeletal conditions that adversely impact the health of older individuals. While both conditions appear to be distinct, the coexistence of the two conditions, known as osteosarcopenia, may identify a subpopulation of older individuals at greater risk of adverse health outcomes compared to either condition alone. Osteosarcopenia is associated with a 3- to 4-fold increased odds of falls and fractures in older individuals attending a falls clinic [1]. Osteosarcopenic individuals who suffered a hip fracture also had 1.8 times greater hazard for mortality compared to non-osteosarcopenic individuals [2].

Despite some studies demonstrating the deleterious effects of osteosarcopenia, others show inconsistent outcomes. For example, community-dwelling older Australian men do not have increased falls, fractures or mortality risk above and beyond either condition alone [3,4]. These discrepant findings may be ascribed to differences in the population under study and the chosen outcomes. In a cohort of independent community-dwelling older individuals, frailty is a particularly salient outcome, representing increased vulnerability to adverse events. However, few studies have examined the association between osteosarcopenia and frailty [5,6,7], relative to osteoporosis and sarcopenia alone.

Nutrition plays a key role in the pathogenesis of frailty, and it is also important in both bone [8] and muscle health [9]. However, the complex relationships between osteosarcopenia, nutrition and frailty remain to be elucidated. Specifically, the mediating role of nutrition in the relationship between osteosarcopenia and frailty bears further scrutiny. Clarifying this relationship will explicate the role of nutrition as a mechanism through which osteosarcopenia engenders frailty, and establish nutrition as an appropriate target for intervention in preventing the adverse consequences of osteosarcopenia.

Therefore, we aimed to, firstly, examine the association between osteosarcopenia, osteoporosis and sarcopenia with frailty and markers of nutritional status. Secondly, we investigated nutrition as a mediator of the relationship between osteosarcopenia and frailty, relative to osteoporosis and sarcopenia alone, in a cohort of independent community-dwelling older adults. We hypothesize that osteosarcopenia is associated with frailty and markers of poor nutritional status, and that the relationship between osteosarcopenia and frailty is mediated by nutrition.

## 2. Materials and Methods

### 2.1. Participants and Setting

This is a cross-sectional analysis of 230 participants recruited from the “Longitudinal Assessment of Biomarkers for characterization of early Sarcopenia and Osteosarcopenic Obesity in predicting frailty and functional decline in community-dwelling Asian older adults Study” (GeriLABS 2), a prospective cohort study involving community-dwelling cognitively intact and functionally independent adults aged 50 years and older [10]. Inclusion criteria were: (i) participants aged 50 to 99 years at study enrollment, (ii) community-dwelling, (iii) independent in both activities of daily living (ADLs) and instrumental ADLs, and (iv) non-frail as defined by the FRAIL criteria [11]. We excluded participants with dementia or evidence of cognitive impairment (modified Chinese version of Mini-Mental State Examination (CMMSE) score ≤ 21) [12]; who were unable to walk 8-m independently; or living in a sheltered or nursing home. All participants provided written informed consent to participate in the study. Ethics approval for this study was obtained from the Domain Specific Review Board of the National Healthcare Group (DSRB Ref: 2017/00850).

### 2.2. Data Collection

We collected demographic characteristics (age, gender, ethnicity) and comorbid conditions. Cognitive performance was assessed using the modified Chinese version of Mini-Mental State Examination (CMMSE). Functional status was evaluated using Barthel’s basic activities of daily living (BADL) index [13] and Lawton and Brody’s instrumental ADL (IADL) index [14]. Depressive symptoms were evaluated using the Geriatric Depression Scale (GDS) [15].

Body composition and bone mineral density (BMD) of lumbar spine, femoral neck and total hip of all participants were obtained with the same dual energy X-ray absorptiometry (DXA) source (Discovery^TM^ APEX 13.3; Hologic, Bedford, MA, USA). Osteoporosis was classified by the WHO (World Health Organization) criteria based on the BMD T-score of the lumbar spine and/or femoral neck and/or total hip equivalent or lower than −2.5 SD [16]. Sarcopenia was diagnosed with the Asian Working Group for Sarcopenia (AWGS) 2019 consensus criteria [17], defined as low relative appendicular skeletal muscle mass (ASM) (height-adjusted ASM (ASM/height^2^)) in the presence of either low handgrip strength or slow gait speed using updated cutoffs [18]. Osteosarcopenia was defined as co-existent osteoporosis and sarcopenia.

Physical frailty was assessed using the modified Fried phenotypic criteria [19]. The modified Fried criteria was operationalized as follows [20]: (1) Body mass index less than 18.5; (2) handgrip strength range <28 kg for men and <18 kg for women, measured using a hydraulic hand dynamometer (North Coast Exacta™ Hydraulic Hand Dynamometer; North Coast Medical, Inc., Morgan Hill, CA, USA); (3) usual gait speed < 1.0 m/s on the 3 m walk test; (4) low physical activity defined using the pentile cutoff of ≤29 on the Frenchay Activities Index [21]; and (5) fatigue endorsed on either of two questions from the Center for Epidemiologic Studies–Depression Scale (CES-D) modified to assess fatigue. The five items were added to yield a total score for frailty (range 0–5). Higher scores indicated worsening frailty status.

Nutritional assessments included anthropometric measurements: standing height and body weight were measured to calculate body mass index (BMI), waist circumference (WC), mid-arm circumference (MAC), and calf circumference (CC). WC, MAC and CC were measured using a non-elastic tape. WC was measured at 2.5 cm above the umbilicus. MAC of the dominant arm was obtained with the subject seated, measured at the midpoint between the tip of the shoulder and the tip of the elbow. CC was obtained with the subject in a seated position with both feet on the ground to obtain the maximal reading of the dominant calf [10]. To evaluate nutritional status, the Mini Nutritional Assessment (MNA) [22] was also used. In accordance with established cut-offs, a total MNA score of ≥24 (out of a maximum of 30 points) classifies well-nourished/normal nutritional status, 17 to 23.5 points classifies individuals at risk of malnutrition, and <17 points malnourished. Lastly, serum 25-hydroxyvitamin D (25(OH)D) levels were also obtained.

### 2.3. Statistical Analysis

Continuous variables were expressed as means and standard deviation. Categorical variables were expressed as counts and percentages. One-way analysis of variance (ANOVA) followed by Bonferroni post hoc analyses were used to analyse the relationships of continuous variables (Frailty score and nutritional parameters) with participants classified as osteoporotic, sarcopenic, osteosarcopenic or normal (neither osteoporotic, sarcopenic nor osteosarcopenic) respectively. Multiple linear regression was carried out with frailty score and the aforementioned nutritional parameters as dependent variables, unadjusted and adjusted for age, gender and ethnicity.

**Figure 1:**
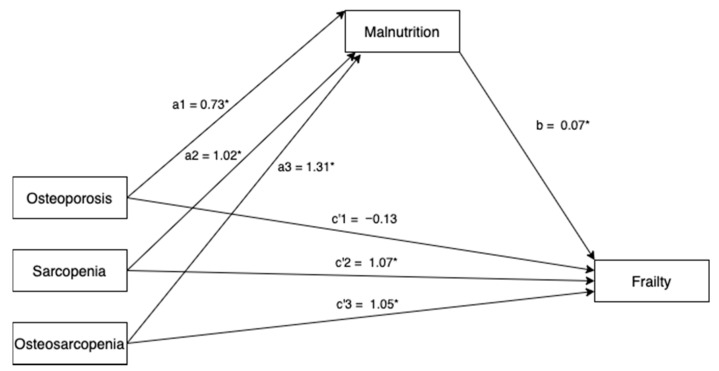
Multicategorical mediation model for total frailty score (* *p* < 0.05). Paths a1, a2, a3 represent the effect of osteoporosis, sarcopenia and osteosarcopenia on the proposed mediator, malnutrition; path b represents the effect of the proposed mediator, malnutrition on frailty; path c’1, c’2 and c’3 represent the relative direct effect of osteoporosis, sarcopenia and osteosarcopenia on frailty. All paths are represented by unstandardized regression coefficients.

A multicategorical mediation model examined the potential mediating role of nutrition, using total MNA score, in the relationship between osteoporosis, sarcopenia and osteosarcopenia with frailty. Dummy codes were created for osteoporosis, sarcopenia and osteosarcopenia with normal individuals (neither osteoporotic, sarcopenic nor osteosarcopenic) as the reference group (Figure 1). Direct and indirect effects for osteoporosis, sarcopenia and osteosarcopenia groups were compared to this reference group. To obtain estimates of the indirect effects, we used bootstrapping procedures based on 5000 resamples [23]. Participants’ age, gender and ethnicity were included in the model as covariates. Two-sided tests with a significance level of *p* < 0.05 were applied. Statistical analysis was carried out using Stata version 13 (StataCorp, College Station, TX, USA).

## 3. Results

### 3.1. Baseline Characteristics

We included 230 individuals (mean age 67.2 ± 7.4 years) in this study. Twenty-seven (11.7%) were osteosarcopenic, 35 (15.2%) were sarcopenic, 36 (15.7%) were osteoporotic and 132 (57.4%) were normal (neither osteosarcopenic, sarcopenic nor osteoporotic). A significant difference in age was observed between the four groups (*p* = 0.0005); post-hoc comparisons with Bonferroni correction revealed that osteosarcopenic (71.4 ± 6.9 years, *p* = 0.001) and sarcopenic individuals (69.3 ± 7.1 years, *p* = 0.048) were significantly older compared to normal individuals (65.7 ± 7.3 years). There were no significant differences observed in gender, comorbidities, ADL function, cognitive function, depressive symptoms and Vitamin D levels between the groups (Table 1).

### 3.2. Association between Osteoporosis, Sarcopenia and Osteosarcopenia with Frailty

There was a statistically significant difference in frailty score between the four groups (*p* < 0.001). Post hoc analyses with Bonferonni correction revealed that frailty score was significantly higher in participants with osteosarcopenia (1.7 ± 0.72, *p* < 0.001) and sarcopenia (1.6 ± 0.81, *p* < 0.001) compared to those who were normal (0.38 ± 0.64). There were no differences in frailty score between osteoporotic-only and normal individuals. There were also no differences in frailty score between osteosarcopenic and sarcopenic individuals (Table 1).

In a multiple linear regression model adjusted for age, gender and ethnicity, sarcopenia (β = 1.15, 95% CI 0.90–1.4, *p* < 0.001) and osteosarcopenia (β = 1.14, 95% CI 0.86–1.4, *p* < 0.001) were associated with frailty, but not osteoporosis (β = −0.077, 95% CI −0.32–0.17, *p* = 0.54) (Table 2).

### 3.3. Association between Osteoporosis, Sarcopenia and Osteosarcopenia with Nutrition

There were statistically significant differences in total MNA score, BMI, WC, MAC and CC between the four groups. MNA total score was significantly lower in osteosarcopenic (26.5 ± 1.9) compared to normal individuals (27.6 ± 1.7, *p* = 0.028). In the whole cohort, using established cutoffs for MNA total score, there were no individuals classified as malnourished, 13 (5.7%) were classified as “at risk” of malnutrition, and 217 (94.4%) were classified as normal. There were no significant differences in MNA categories between the four groups. BMI was significantly lower in osteosarcopenic (22.3 ± 2.5 kg/m^2^, *p* = 0.001), sarcopenic (22.5 ± 2.2 kg/m^2^, *p* = 0.001) and osteoporotic individuals (22.9 ± 2.8 kg/m^2^, *p* = 0.005) compared to normal individuals (24.8 ± 3.4 kg/m^2^). WC and MAC were also lower in osteosarcopenic compared to normal individuals (WC: 81.7 ± 7.7 cm versus 86.9 ± 10.0 cm, *p* = 0.045; MAC: 25.6 ± 2.6 cm versus 28.4 ± 2.8 cm, *p* < 0.001). CC was also significantly lower in osteosarcopenic (32.3 ± 3.6 cm, *p* < 0.001) and sarcopenic individuals (33.3 ± 2.5 cm, *p* < 0.001) compared to normal individuals (35.8 ± 3.0 cm) (Table 1).

Osteosarcopenia was significantly associated with total MNA score, BMI, CC, WC and MAC in multiple linear regression models, unadjusted and adjusted for age, gender and ethnicity. The coefficients observed were of a greater magnitude for individuals with osteosarcopenia, compared to individuals with osteoporosis or sarcopenia alone (Table 3).

### 3.4. Mediating Effect of Nutrition on the Relationship between Osteoporosis, Sarcopenia and Osteosarcopenia with Frailty

In the multicategorical mediation model, there were significant relationships between osteoporosis (a1 coefficient (SE) = −0.73 (0.34), *p* = 0.033), sarcopenia (a2 coefficient (SE) = −1.02 (0.35), *p* = 0.004) and osteosarcopenia (a3 coefficient (SE) = −1.31 (0.39), *p* = 0.001) with total MNA scores. The mediating variable, total MNA score, was significantly related to total frailty score (b coefficient (SE) = −0.07 (0.02), *p* = 0.002). Bootstrapping revealed significant relative indirect effects for osteoporosis (estimate = 0.05, bootstrap 95% CI 0.007–0.14), sarcopenia (estimate = 0.07, bootstrap 95% CI 0.02–0.17) and osteosarcopenia (estimate = 0.09, bootstrap 95% CI 0.01–0.2), indicating that nutrition mediated the association between osteoporosis, sarcopenia and osteosarcopenia, relative to normal individuals. The proportion of the total effect, mediated by nutrition, is 10% (Table 4).

## 4. Discussion

Our study demonstrated that in relatively healthy community-dwelling older adults, osteosarcopenia is present in 11.7% of individuals and is associated with markers of nutrition, including lower MNA scores, BMI, calf, mid-arm and waist circumference. In the association between osteosarcopenia, sarcopenia and osteoporosis with frailty, we observed a mediating effect of nutrition, which was stronger in osteosarcopenic individuals compared to individuals with sarcopenia or osteoporosis alone.

The prevalence of osteosarcopenia in our study lies within previous estimates in Asian populations, which ranges from 6.2% [24] to 19.6% [25]. In contrast, in individuals attending falls clinics or with osteoporotic fractures, prevalence estimates are substantially higher, ranging up to 58% [26]. These differences in the population studied may account for inconsistent associations between osteosarcopenia and negative outcomes.

In our cohort of relatively younger, independent community-dwelling individuals, we found a significant association between sarcopenia and frailty, with and without concomitant osteoporosis. Both sarcopenia and osteoporosis contribute to frailty through common pathways, including chronic inflammation, which exacerbates muscle [27] and bone loss [28]. Mechanisms include the accumulation and altered distribution of body fat into intra- and intermuscular adipose tissue [29], altering lipid metabolism and promoting the accumulation of macrophages and other immune cells, and increasing the secretion of proinflammatory cytokines [30]. Together, these contribute to a state of chronic, low-grade inflammation favoring the development and progression of sarcopenia. Similarly, in older women, higher levels of inflammatory markers (soluble interleukin-6, tumor necrosis factor receptors 1 and 2) were associated with increased risk of hip fractures, and this association was mediated by BMD [31], further supporting the role of inflammation in both sarcopenia and osteoporosis.

Metabolic and hormonal pathways, particularly in the growth hormone (GH)/insulin-like growth factor-1 (IGF-1) axis, are essential for metabolic regulation of bone and muscle mass, with the decline in GH and IGF-1 levels associated with both osteoporosis and sarcopenia [32]. The dysregulation of this pathway with ageing may also play a role in the pathogenesis of frailty [20], with a study showing an independent association of low levels of IGF-1 with pre-frailty and frailty [33]. Nevertheless, we did not find any associations between osteoporosis alone and frailty. Furthermore, the strength of the association between osteosarcopenia and frailty was also similar to that of sarcopenia and frailty, highlighting the relative importance of interventions to improve muscle health in this cohort to mitigate frailty.

The present study reveals an association between osteosarcopenia, sarcopenia and osteoporosis with markers of nutritional status, with poorer nutritional status observed with osteosarcopenia, compared to isolated sarcopenia and osteoporosis. These findings are consistent with a previous study of geriatric inpatients [34] and individuals attending a falls and fractures clinic [35]. Our finding that nutrition mediates the association between osteosarcopenia and frailty further underscores the crucial role of nutrition in the development of frailty in at-risk individuals with osteosarcopenia. Specifically, loss of muscle mass and increased risk of osteoporosis are associated with caloric deficit, protein and Vitamin D deficiency, which in turn are modifiable nutritional risk factors for frailty [36]. Thus, our findings further provide the impetus for nutritional assessment and early supplementation in osteosarcopenia to ameliorate the detrimental effects of frailty. However, in this cohort, the proportion of the total effect mediated by nutrition is small, suggesting that other mechanisms, such as physical activity or social factors [37], are also important to prevent the development of frailty.

In our cohort of relatively healthy, independent, community-dwelling older adults, there were no individuals classified as malnourished using established cutoffs on the MNA total score. Items in the MNA also overlap with components included in definitions of sarcopenia and frailty, although the effect of the potential “circularity” is likely to be limited, as the R^2^ values in the unadjusted regression models for BMI, CC and MAC are not large. Despite these observations, our results still demonstrate the associations between osteosarcopenia, malnutrition and frailty, albeit with malnutrition having only a small mediating effect on this association. A possible explanation is that the MNA does not provide accurate quantification of protein, caloric and micronutrient intake relevant to bone and muscle health, but instead provides a global assessment of malnutrition risk. This suggests that in independent community-dwelling older adults presenting upstream of the spectrum of frailty, developing nutritional screening tools which are more sensitive to milder degrees of malnutrition (e.g., detection of anorexia as a forward indicator of malnutrition) [38], or with components specific to bone and muscle health may better identify individuals at risk of osteosarcopenia, and subsequent development of frailty.

The strengths of this study include the analysis of a reasonably-sized cohort of independent community-dwelling older adults, the use of contemporary consensus definitions for sarcopenia in an Asian population, and comprehensive evaluation of nutritional status. To our knowledge, this is the first study to use mediation analysis to explicate nutrition as a mechanism underlying the link between osteosarcopenia and frailty. Weaknesses include the cross-sectional nature of this study, which preclude definitive conclusions about causality. The proposed pathway is also exploratory and not exhaustive, and needs to be corroborated in well-designed longitudinal studies. The possibility of residual confounding factors, such as physical activity, which was not measured in this study, also exists. Lastly, we are unable to generalize our results to other older, frailer populations.

In summary, the present study showed that sarcopenia, with and without osteoporosis, is associated with increased frailty, and that nutrition mediates the association between osteosarcopenia and frailty. These findings are salient to older adults living amidst the COVID-19 pandemic, as the need for physical distancing and social isolation may adversely impact nutritional status [39]. Taken together, our results support the need for early identification and interventions for malnutrition in at-risk older adults with osteosarcopenia to prevent the downstream cascade of frailty and disability.

## Figures and Tables

**Table 1:** Baseline characteristics, nutritional and frailty status.

Characteristics	Normal (*N* = 132)	O (*N* = 36)	S (*N* = 35)	OS (*N* = 27)	*p*
Age in years, mean (SD)	65.7 (7.3)	67.6 (7.0)	69.3 (7.1) *	71.4 (6.9) *	0.0005
Female gender, N (%)	99 (75)	30 (83.3)	23 (65.7)	15 (55.6)	0.064
Chinese ethnicity, N (%)	116 (87.9)	36 (100)	35 (100)	25 (92.6)	0.024
Comorbidities, N (%)					
Hypertension	44 (33.3)	13 (36.1)	15 (42.9)	10 (37.0)	0.77
Hyperlipidemia	69 (52.3)	23 (63.9)	19 (54.3)	19 (70.4)	0.27
Diabetes mellitus	13 (9.9)	7 (19.4)	8 (22.9)	5 (15.8)	0.15
Ischemic heart disease	3 (2.3)	1 (2.8)	1 (2.9)	0	0.86
Stroke or transient ischemic attack	3 (2.3)	0	1 (2.9)	0	0.66
Kidney disease	7 (5.3)	0	2 (5.7)	0	0.31
Asthma	10 (7.6)	1 (2.8)	3 (8.6)	1 (3.7)	0.65
Arthritis	16 (12.1)	1 (2.8)	5 (14.3)	3 (11.1)	0.38
Physical function, mean (SD)					
ADL	98.0 (3.3)	98.9 (3.0)	98.3 (3.0)	97.4 (4.2)	0.31
Cognitive function, mean (SD)					
CMMSE	26.3 (1.7)	25.9 (1.8)	25.7 (1.8)	26.2 (1.8)	0.30
Depressive symptoms, mean (SD)					
GDS	1.1 (1.8)	1.1 (2.0)	1.2 (1.1)	1.2 (0.9)	0.99
Nutrition and anthropometry, mean (SD)					
MNA total score	27.6 (1.7)	26.8 (1.9)	26.7 (2.0)	26.5 (1.9) *	0.0035
MNA categories, N (%)					0.22
At risk	4 (3.0)	4 (11.1)	3 (8.6)	2 (7.4)	
Normal	128 (97.0)	32 (88.9)	32 (91.4)	25 (92.6)	
BMI (kg/m^2^)	24.8 (3.4)	22.9 (2.8) *	22.5 (2.2) *	22.3 (2.5) *	<0.001
Calf circumference (cm)	35.8 (3.0)	34.5 (2.7)	33.3 (2.5) *	32.3 (3.6) */**	<0.001
Mid-arm circumference (cm)	28.4 (2.8)	26.9 (2.9)	27.0 (3.0) *	25.6 (2.6) */**	<0.001
Waist circumference (cm)	86.9 (10.0)	83.2 (8.9)	84.2 (7.0)	81.7 (7.7) *	0.015
DEXA fat%	38.8 (6.2)	38.2 (6.0)	37.5 (7.2)	36.1 (7.6)	0.22
Serum Vitamin D levels (μg/L)	29.9 (8.5)	30.4 (80)	31.9 (10.2)	32.7 (9.3)	0.38
Fried Frailty total score, mean (SD)	0.38 (0.64)	0.33 (0.68)	1.6 (0.81) */**	1.7 (0.72) */**	<0.001

O, osteoporosis; S, sarcopenia; OS, osteosarcopenia; SD, standard deviation; ADL, activities of daily living; CMMSE, modified version of the Chinese Mini-Mental State Examination; GDS, Geriatric Depression Scale; MNA, Mini-Nutritional Assessment; BMI, body mass index; DEXA, dual-energy x-ray absorptiometry. * *p* < 0.05 vs. normal; ** *p* < 0.05 vs. osteoporosis.

**Table 2:** Multiple linear regression models with total frailty score as the dependent variable.

	Unadjusted Model				Adjusted Model *			
Total Frailty Score	β	SE	95% CI	*p*	β	SE	95% CI	*p*
Osteoporosis	−0.045	0.13	−0.30–0.21	0.72	−0.077	0.12	−0.32–0.17	0.54
Sarcopenia	1.25	0.13	0.99–1.50	<0.001	1.15	0.13	0.90–1.40	<0.001
Osteosarcopenia	1.32	0.14	1.04–1.61	<0.001	1.14	0.14	0.86-1.42	<0.001
	*R*^2^ = 0.057				*R*^2^ = 0.50			

SE, standard error; CI, confidence interval. * Adjusted for age, gender and ethnicity.

**Table 3:** Multiple linear regression models with nutritional parameters as dependent variables.

Unadjusted Models	MNA (Total Score)			BMI, kg/m^2^			Calf Circumference, cm			Waist Circumference, cm			Mid-Arm Circumference, cm		
	**β**	**SE**	***p***	**β**	**SE**	***p***	**β**	**SE**	***p***	**β**	**SE**	***p***	**β**	**SE**	***p***
Osteoporosis	−0.73	0.34	0.035	−1.95	0.57	0.001	−1.25	0.56	0.026	−3.71	1.72	0.032	−1.52	0.54	0.005
Sarcopenia	−0.89	0.35	0.011	−2.3	0.58	<0.001	−2.43	0.56	<0.001	−2.75	1.74	0.116	−1.4	0.54	0.011
Osteosarcopenia	−1.1	0.39	0.005	−2.57	0.64	<0.001	3.49	0.62	<0.001	−5.22	1.94	0.008	−2.84	0.6	<0.001
	R^2^ = 0.058			R^2^ = 0.12			R^2^ = 0.16			R^2^ = 0.045			R^2^ = 0.11		
Adjusted Models *	**MNA (Total Score)**			**BMI, kg/m^2^**			**Calf Circumference, cm**			**Waist Circumference, cm**			**Mid-Arm Circumference, cm**		
****	**β**	**SE**	***p***	**β**	**SE**	***p***	**β**	**SE**	***p***	**β**	**SE**	***p***	**β**	**SE**	***p***
Osteoporosis	−0.73	0.35	0.037	−1.94	0.57	0.001	−1.07	0.56	0.057	−3.05	1.64	0.063	−1.44	0.54	0.008
Sarcopenia	−1.02	0.36	0.005	−2.39	0.59	<0.001	−2.44	0.57	<0.001	−3.36	1.67	0.046	−1.52	0.56	0.007
Osteosarcopenia	−1.31	0.4	0.001	−2.91	0.66	<0.001	−3.65	0.64	<0.001	−7.24	1.87	<0.001	−3.12	0.62	<0.001
	R^2^ = 0.082			R^2^ = 0.16			R^2^ = 0.19			R^2^ = 0.18			R^2^ = 0.13		

SE, standard error; CI, confidence interval. * Adjusted for age, gender and ethnicity.

**Table 4:** Multicategorical mediation model: relative direct, total and indirect effects for the mediating role of nutrition on the relationship between osteosarcopenia, osteoporosis and sarcopenia with frailty.

				Coefficient (SE)				*p*
Osteoporosis								
a1				−0.73 (0.34)				0.033
Sarcopenia								
a2				−1.02 (0.35)				0.004
Osteosarcopenia								
a3				−1.31 (0.39)				0.001
	**Relative Total Effects**			**Relative Direct Effects**			**Relative Indirect Effects**	
	**Coefficient (SE)**	***p***		**Coefficient (SE)**	***p***		**Coefficient (SE)**	**95% CI**
c1	−0.08 (0.12)	0.54	c’1	−0.13 (0.12)	0.28	a1b	0.05 (0.03)	0.007–0.14
c2	1.15 (0.13)	<0.001	c’2	1.07 (0.12)	<0.001	a2b	0.07 (0.04)	0.02–0.17
c3	1.14 (0.14)	<0.001	c’3	1.05 (0.14)	<0.001	a2c	0.09 (0.05)	0.01–0.22
			b	−0.07 (0.02)	0.002			
